# A randomized controlled trial to determine whether beta-hydroxy-beta-methylbutyrate and/or eicosapentaenoic acid improves diaphragm and quadriceps strength in critically Ill mechanically ventilated patients

**DOI:** 10.1186/s13054-021-03737-9

**Published:** 2021-08-26

**Authors:** Gerald S. Supinski, Paul F. Netzel, Philip M. Westgate, Elizabeth A. Schroder, Lin Wang, Leigh Ann Callahan

**Affiliations:** 1 Division of Pulmonary, Critical Care and Sleep Medicine, Department of Internal Medicine, College of Medicine, University of Kentucky, 740 South Limestone, L543, Lexington, KY 40536-0284 USA; 2 Department of Biostatistics, College of Public Health, University of Kentucky, 725 Rose Street, Lexington, KY MDS 205B40536-0082 USA

**Keywords:** Diaphragm weakness, Respiratory failure, Mechanical ventilation, Limb muscle weakness, Beta-hydroxy-beta-methylbutyrate, Eicosapentaenoic acid

## Abstract

**Background:**

Intensive care unit acquired weakness is a serious problem, contributing to respiratory failure and reductions in ambulation. Currently, there is no pharmacological therapy for this condition. Studies indicate, however, that both beta-hydroxy-beta-methylbutyrate (HMB) and eicosapentaenoic acid (EPA) increase muscle function in patients with cancer and in older adults. The purpose of this study was to determine whether HMB and/or EPA administration would increase diaphragm and quadriceps strength in mechanically ventilated patients.

**Methods:**

Studies were performed on 83 mechanically ventilated patients who were recruited from the Medical Intensive Care Units at the University of Kentucky. Diaphragm strength was assessed as the trans-diaphragmatic pressure generated by supramaximal magnetic phrenic nerve stimulation (PdiTw). Quadriceps strength was assessed as leg force generated by supramaximal magnetic femoral nerve stimulation (QuadTw). Diaphragm and quadriceps thickness were assessed by ultrasound. Baseline measurements of muscle strength and size were performed, and patients were then randomized to one of four treatment groups (placebo, HMB 3 gm/day, EPA 2 gm/day and HMB plus EPA). Strength and size measurements were repeated 11 days after study entry. ANCOVA statistical testing was used to compare variables across the four experimental groups.

**Results:**

Treatments failed to increase the strength and thickness of either the diaphragm or quadriceps when compared to placebo. In addition, treatments also failed to decrease the duration of mechanical ventilation after study entry.

**Conclusions:**

These results indicate that a 10-day course of HMB and/or EPA does not improve skeletal muscle strength in critically ill mechanically ventilated patients. These findings also confirm previous reports that diaphragm and leg strength in these patients are profoundly low. Additional studies will be needed to examine the effects of other anabolic agents and innovative forms of physical therapy.

*Trial registration*: ClinicalTrials.gov, NCT01270516. Registered 5 January 2011, https://clinicaltrials.gov/ct2/show/NCT01270516?term=Supinski&draw=2&rank=4.

**Supplementary Information:**

The online version contains supplementary material available at 10.1186/s13054-021-03737-9.

## Background

Critically ill patients develop significant muscle weakness of both the diaphragm, the major muscle of breathing, and limb muscles. Diaphragm weakness influences intensive care unit (ICU) outcomes, such that patients with weak diaphragms have a much higher mortality and a longer duration of mechanical ventilation than patients with stronger diaphragms [1–6]. In addition, limb muscle weakness has negative consequences for critically ill patients [7, 8]. Limb muscle weakness persists long after ICU discharge, reduces long-term exercise performance, and decreases the ability of patients to perform activities of daily living [7–12].

Importantly, recent studies indicate that both beta-hydroxy-beta-methylbutyrate (HMB) and eicosapentaenoic acid (EPA) improve muscle function in cancer patients and in older adults [13–16]. HMB is thought to exert these effects by inhibition of protein degradation, increases in protein synthesis, and reductions in muscle cell apoptosis, while EPA is thought to reduce protein degradation and to increase mitochondrial biogenesis [17, 18]. There has been, however, only limited study of the effects on these agents on muscle properties in the critically ill [19, 20] and no previous examination of the effects on strength of intensive care unit patients.

The purpose of the present study, therefore, was to determine whether HMB and/or EPA administration would also increase diaphragm and quadriceps strength in mechanically ventilated patients. To accomplish this task, we measured both diaphragm and quadriceps strength by determining, respectively, pressure and force generation in response to supramaximal magnetic stimulation of the nerves (phrenic and femoral) to these muscles in a cohort of mechanically ventilated ICU patients. In addition, we used ultrasound to assess the thickness of the diaphragm and the quadriceps (i.e. the rectus femoris and vastus intermedius). Baseline measurements of muscle strength and size were performed; then patients were randomized to one of four treatment groups (placebo, HMB 3 g/day, EPA 2 g/day and HMB plus EPA). Strength and size measurements were repeated 11 days after study entry. The primary endpoint of the study was to determine whether HMB, EPA, or the combination of these agents improved diaphragm or quadriceps muscle strength. Secondary endpoints were to determine whether these agents increased diaphragm and quadriceps muscle thickness, and/or reduced the time required to wean patients from mechanical ventilation.

## Methods

### Patient selection

Approval to conduct this research was obtained from the University of Kentucky Institutional Review Board (IRB). Consent for inclusion in these studies was obtained from all subjects and/or their surrogates. Inclusion into the study was considered for all adult patients requiring mechanical ventilation for more than 48 h for respiratory failure in one of the University of Kentucky adult medical ICUs. Subjects were included regardless of sex, race, or age. Subjects were excluded: (a) if the physician caring for the patient determined that the patient was too unstable to tolerate these measurements; or if the patient; (b) was receiving high dose pressors (more than 15 mcg/min of norepinephrine or more than 15 mg/kg/min of dopamine), (c) required > 80% FiO_2_ or > 15 cm H_2_O of PEEP, (d) had a cardiac pacemaker or implanted defibrillator, (e) received neuromuscular blocking agents within the 48 h preceding testing, (f) had a history of a preexisting neuromuscular disease, (g) had recent variceal bleeding, (h) was pregnant, (i) was incarcerated, (j) was institutionalized, or (k) if the attending physician thought that the patient was terminal and would have care withdrawn within 7 days.

### Study protocol

After informed consent was obtained, we:

- Measured magnetic stimulated diaphragm twitch pressure (PdiTw), quadriceps twitch strength (QuadTw), diaphragm thickness, quadriceps thickness, lung mechanics (respiratory system compliance, airway resistance), and performed a chart review,
- Randomized patients to enteral treatment with either: placebo (30 ml of salt water solution, 1.5 g of amino acids and 1 ml of corn oil every 12 h), eicosapentaenoic acid (EPA; 30 ml of salt water, 1.5 g of amino acids and 1000 mg EPA every 12 h), beta-hydroxy-beta-methylbutyrate (HMB; 30 ml of salt water solution, 1500 mg HMB and 2 ml of corn oil every 12 h), or both HMB and EPA (30 ml of salt water, 1500 mg HMB and 1000 mg EPA every 12 h).
- Continued placebo or drug treatments for 10 days, and
- On day 11, repeated measurements of PdiTw, QuadTw, diaphragm thickness, and quadriceps thickness.

Randomization of patients was performed by the Research Pharmacy Service at the University of Kentucky following a randomization design formulated by the study statistician (Dr. Westgate). As part of this randomization, patients were stratified by age so that approximately equal numbers of patients greater than 55 years of age and less than 55 years of age would be placed into the four treatment arms. The Research Pharmacy service then arranged for delivery of the drugs to the critical care services caring for patients. Nurses administering the drugs and all investigators obtaining consent and making study measurements were blinded as to patient treatment arm assignments: this blinding continued for the duration of the study.

There are several reasons we chose a course of 10 days of therapy with HMB and/or EPA. First, animal experiments suggest that even short courses of these medications (48 h) should be effective [28, 29], so we thought that extremely long treatments would not be needed. Second, the majority of patients in our hospital remain in the ICU for approximately 10–14 days and many are transferred to long-term ventilator units after this time point if they cannot be weaned from mechanical ventilation. For this reason, we thought it was important that a 10 day course of therapy would be effective; if a drug treatment takes substantially longer than 10 days to improve muscle function, we do not believe it would change the trajectory of mechanical ventilation use in most ICUs in the United States.

In accordance with the IRB approved protocol, research personnel did not make any clinical decisions regarding the management of patients while being treated in the intensive care unit.

### Determination of transdiaphragmatic twitch pressure generation (PdiTw)

Diaphragm strength was assessed by determining transdiaphragmatic twitch pressure (PdiTw) in response to bilateral anterior magnetic stimulation of the phrenic nerves (BAMPS) as previously described in detail [1, 2]. In brief, two sterile balloon tipped catheters (Cooper Surgical, Turnbull, CT) were passed through the nose and one catheter was placed in the stomach and the other placed in the esophagus [1, 2]. Patients were then allowed to breathe quietly for 10 min. Magnetic coils attached to MagStim 200 stimulators were placed bilaterally over the phrenic nerves, and simultaneous supramaximal pulses were delivered to elicit twitch transdiaphragmatic pressure (i.e. PdiTw), while transiently occluding the circuit connecting the endotracheal tube to the ventilator. A minimum of five twitches were recorded at 100% field stimulation, and additional twitches were performed at reduced magnetic field strengths (60–90%). PdiTw was recorded as the average of the best three measurements in response to 100% levels of magnetic stimulation. All the measurements of PdiTw were carried out by Dr. Supinski and Dr. Netzel.

### Diaphragm thickness

Right hemidiaphragm thickness was measured over the zone of apposition using two dimensional B mode and M mode ultrasound and a high-frequency linear array probe (10 MHz) [21]. The transducer was positioned over the mid-axillary line between the 7th and 8th ribs or 8th and 9th ribs, and respiratory variation in diaphragm thickness was assessed by recording diaphragm signals in M mode using the ultrasound caliper utility to measure diaphragm thickness at end expiration. Average end expiratory diaphragm thickness was calculated from three sets of measurements. All the measurements of diaphragm thickness were carried out by Dr. Supinski and Dr. Netzel.

### Measurement of quadriceps twitch strength (QuadTw)

Quadriceps twitch force was measured using magnetic stimulation of the femoral nerve using modifications as previously reported in detail [22, 23]. Prior to measurement, patient remained inactive in bed for 20 min. A quadriceps support was placed under the right leg, positioned with the knee immediately over the apex of the apparatus. To record force, a transducer (Omega Engineering, Inc., Norwalk, CT) was placed around the lower leg at the ankle. A figure of eight coil was placed over the femoral nerve, with the coil powered by a MagStim 200 stimulator (Jali Industries, Ma USA). Coil placement was adjusted to achieve maximum force generation, followed by sequentially stimulating at field strengths between 60 and 100%. QuadTw was recorded as the average of the best three measurements in response to 100% levels of magnetic stimulation. All the measurements of quadriceps force were carried out by Dr. Supinski and Dr. Netzel.

### Quadriceps thickness

We determined right quadriceps thickness by measuring the thickness of the vastus intermedius (VIM) and rectus femoris (RF) at its widest extent (i.e. in the anterior-to posterior orientation, determined as the distance from the adipose tissue-muscle interface to the intermuscular interface for each muscle). The measurements were assessed at 50% of the distance between the lateral condyle of the femur and the greater trochanter. Total quadriceps thickness was recorded as the average of the sum of VIM and RF from the three best measurements obtained. All the measurements of quadriceps thickness were carried out by Dr. Supinski and Dr. Netzel.

### Clinical parameters

Clinical parameters including: age, gender, diagnoses, vital signs, duration of mechanical ventilation prior to PdiTw measurement, mechanical ventilation mode, FiO_2_, tidal volume and rate, and most recent arterial blood gas values, were collected as close as possible to the time of determination of PdiTw and QuadTw. We also recorded the sequential organ failure assessment (SOFA) score and Charlson Comorbidity Score (CCS) for each patient. Finally, since the time required to wean patients from mechanical ventilation is thought to be influenced by respiratory muscle function [1, 2, 5, 6], we also assessed the effect of the various treatment regimens on the duration of mechanical ventilation after study entry.

### Nutrition, physical therapy, patient sedation, and mechanical ventilation weaning regimens

All the patients in this study were provided physical therapy and occupational therapy in keeping with the standard regimens employed by the physical and occupational therapy department of the University of Kentucky hospitals. In addition to these sessions, bedside nurses provided range-of-motion exercises to patients each shift. Detailed descriptions of the physical therapy protocols, as well as the nutritional protocol and mechanical ventilation weaning protocol, are provided in the Additional file 1 (Methods.docx).

### Statistical analysis

A power analysis performed prior to initiation of the study estimated that we needed approximately 18 patients/group (total of 72) to detect a 30% improvement in muscle strength between the placebo group and a drug treatment group with an assumed standard deviation of 24.5%, a power of 0.85, and an alpha level of 0.05. Subject recruitment was ended when we achieved initial measurements of strength in 73 patients.

Baseline comparisons (Tables 1 and 2) were conducted via one-way ANOVA. ANCOVA testing was used to compare changes in key variables measured before and after drug treatment for the four experimental groups, including diaphragm twitch (natural log), diaphragm thickness (natural log), quadriceps force and quadriceps thickness, with correction for the corresponding initial measure and several potential confounding variables (age, sex, Charlson Comorbidity Score, Sequential Organ Failure Score (SOFA), duration of mechanical ventilation prior to randomization into the study). A similar analysis was conducted to compare the natural log of mechanical ventilation durations. A p value of less than 0.05 was taken as indicating statistical significance for comparisons. Analyses were conducted in SAS version 9.4 (SAS Institute, Cary, NC, USA).

**Table 1:** Patient demographics

	Control	EPA	HMB	HMB + EPA	*P* values
	(*n* = *20*)	(*n* = *17*)	(*n* = *18*)	(*n* = *18*)	
	Mean	Mean	Mean	Mean	
	(*95% CI*)	(*95% CI*)	(*95% CI*)	(*95% CI*)	
Age (years)	**55.5**	**52.9**	**61.2**	**56.2**	**0.384**
	(*47.7–63.1*)	(*46.0–59.7*)	(*54.0–68.4*)	(*49.7–62.7*)	
Weight (kg)	**86.5**	**89.3**	**93.7**	**83.8**	**0.714**
	(*76.0–96.9*)	(*75.8–102.8*)	(*81.3–106.0*)	(*67.6–99.9*)	
SOFA	**7.0**	**7.7**	**6.7**	**8.1**	**0.619**
	(*5.3–8.7*)	(*6.0–9.4*)	(*5.3–8.1*)	(*6.1–10.1*)	
CCS	**4.0**	**3.0**	**3.6**	**2.9**	**0.483**
	(*2.7–5.3*)	(*1.6–4.4*)	(*2.4–4.7*)	(*1.9–3.9*)	
Prior MV duration (days)	**9.2**	**8.8**	**7.5**	**7.3**	**0.875**
	(*4.7–13.6*)	(*5.1–12.4*)	(*4.0–11.0*)	(*3.1–11.5*)	
Systolic BP (mm Hg)	**119**	**115**	**120**	**119**	**0.836**
	(*111–127*)	(*106–124*)	(*112–129*)	(*110–128*)	
Diastolic BP (mm Hg)	**68**	**61**	**63**	**68**	**0.344**
	(*61–74*)	(*53–69*)	(*56–70*)	(*62–73*)	
Heart rate (beats/min)	**87.3**	**90.9**	**82.4**	**93.6**	**0.264**
	(*78.5–96.0*)	(*81.8–100.1*)	(*74.4–90.5*)	(*84.9–102.2*)	
Temp (°F)	**99.1**	**99.2**	**99.2**	**98.4**	**0.134**
	(*98.5–99.8*)	(*98.6–99.8*)	(*98.5–99.9*)	(*97.8–98.9*)	
Respirations (breaths/min)	**22.3**	**23.1**	**20.0**	**21.5**	**0.289**
	(*19.4–25.1*)	(*20.2–26.1*)	(*17.2–22.1*)	(*18.9–24.1*)	
FiO_2_	**0.43**	**0.52**	**0.44**	**0.45**	**0.149**
	(*0.39–0.48*)	(*0.44–0.58*)	(*0.38–0.49*)	(*0.41–0.49*)	
PEEP (cm H_2_O)	**5.9**	**8.3**	**6.2**	**6.4**	**0.056**
	(*5.1–6.7*)	(*6.2–10.4*)	(*4.9–7.5*)	(*5.1–7.8*)	
Static compliance^a^	**26.0**	**23.0**	**23.2**	**24.0**	**0.673**
	(*21.3–30.8*)	(*19.1–26.9*)	(*20.1–26.3*)	(*19.5–28.4*)	
Airway resistance^b^	**10.7**	**10.0**	**14.8**	**12.5**	**0.128**
	(*8.7–12.7*)	(*6.9–13.1*)	(*10.1–19.5*)	(*9.9–15.2*)	
Number of doses	**19.0**	**18.2**	**17.8**	**17.0**	**0.465**
	(*18.1–19.9*)	(*15.7–20.7*)	(*15.9–19.7*)	(*14.8–19.2*)	
Number of PT-OT sessions	**2.6**	**2.6**	**1.8**	**2.9**	**0.590**
	(*1.3–3.9*)	(*1.5–3.7*)	(*0.8–2.8*)	(*1.4–4.3*)	
Primary	Enteral	Enteral	Enteral	Enteral	
Nutrition route	Tube feeding	Tube feeding	Tube feeding	Tube feeding	
Degree of dependence	Independent 55%	Independent 59%	Independent 61%	Independent 67%	
	Assistance 45%	Assistance 41%	Assistance 39%	Assistance 33%	
Main diagnoses	Respiratory failure 35%	Respiratory failure 53%	Respiratory failure 56%	Respiratory failure 50%	
	Sepsis 45%	Sepsis 41%	Sepsis 44%	Sepsis 56%	
	Pneumonia 60%	Pneumonia 53%	Pneumonia 33%	Pneumonia 39%	

Bold was used to highlight the groups, means and *P* values

Italics represents the *n* = number of subjects per group and the 95% confidence interval

CI = Confidence interval, SOFA = sequential organ failure score, CCS = Charlson comorbidity score, MV = mechanical ventilation, BP = blood pressure, mm Hg = millimeters of mercury, F = Fahrenheit, FiO_2_ = fraction of inspired oxygen, PEEP = positive end expiratory pressure, H_2_O = water, PT = physical therapy, OT = occupational therapy

^a^Respiratory system static compliance (ml/cm H_2_O)

^b^Airway resistance (cm H_2_O/liter/second)

**Table 2:** Initial patient diaphragm and quadriceps characteristics

	Control	EPA	HMB	HMB + EPA	
	Mean	Mean	Mean	Mean	*P* values
	(*95% CI*)	(*95% CI*)	(*95% CI*)	(*95% CI*)	
	*n*	*n*	*n*	*n*	
Diaphragm strength (PdiTw)	**7.4**	**5.4**	**3.7**	**5.8**	**0.050**
	*4.7–10.1*	*4.2–6.5*	*2.5–4.9*	*4.0–7.6*	
	*n* = 19	*n* = 15	*n* = 15	*n* = 15	
Diaphragm thickness (cm)	**0.26**	**0.27**	**0.26**	**0.25**	**0.878**
	*0.23–0.29*	*0.24–0.30*	*0.23–0.30*	*0.22–0.28*	
	*n* = 19	*n* = 15	*n* = 16	*n* = 17	
Quadriceps strength (QuadTw)	**5.9**	**5.2**	**4.6**	**5.1**	**0.913**
	*3.1–8.6*	*1.9–8.6*	*2.8–6.4*	*3.0–7.3*	
	*n* = 20	*n* = 17	*n* = 16	*n* = 18	
Quadriceps thickness (cm)	**2.8**	**3.3**	**3.1**	**2.6**	**0.327**
	*2.4–3.3*	*2.7–3.8*	*2.6–3.6*	*1.9–3.3*	
	*n* = 20	*n* = 16	*n* = 15	*n* = 18	

Bold was used to highlight the groups, means and *P* values

Italics represents the *n* = number of subjects per group and the 95% confidence interval

CI = Confidence interval, PdiTw shown in cm H_2_O, QuadTw in Newtons, cm = centimeters, n = number of measurements per treatment arm-as indicated elsewhere in the text, every measurement could not be obtained from each subject. Data represent actual values obtained for each measurement

## Results

### Patient characteristics

**Fig. 1:**
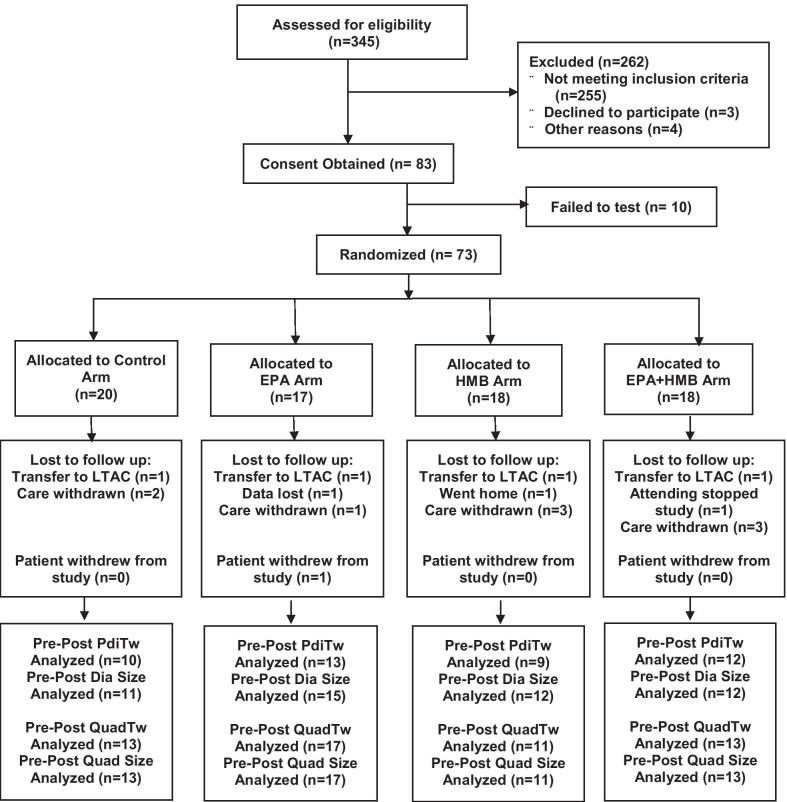
Consort diagram. We initially screened 345 patients and consent was obtained from 83. Of these, 10 failed to complete initial measurements of diaphragm size and strength, with the remaining 73 patients randomized to the four treatment arms. Several patients were lost or withdrew during the treatment administration phase in each study group (i.e. 3 in the control (placebo), 4 in the EPA, 5 in the HMB and 5 in the HMB + EPA arm) due to death, transfer, or request to be removed from the study. In the remaining patients, we obtained data examining changes in diaphragm strength (PdiTw), diaphragm size, quadriceps strength (QuadTw) and quadriceps size between initial, pre-treatment and final, post-treatment measurements

We assessed 345 patients for study eligibility of which 262 were excluded because of failure to meet study inclusion and exclusion criteria (see Fig. 1). Consent was obtained in the remaining 83 patients. Not every parameter could be successfully completed in each subject. Ten patients either withdrew before measurements could be made or there were technical issues (e.g. massive obesity) that precluded any measurement of diaphragm and quadriceps strength. Adequate measurements of initial diaphragm or quadriceps parameters were made in the remaining 73 patients but technical issues prevented acquisition of complete data sets in several patients. Technically adequate (i.e. achieving supramaximal twitches) baseline diaphragm strength measurements (PdiTw) were obtained in 64 of 73 patients and technically adequate baseline quadriceps strength (QuadTw) were achieved in 71 of 73 patients. As a result, the present report includes baseline PdiTw data for 64 patients and baseline QuadTw data for 71 patients.

Patients recruited to this study were severely ill, with mean sequential organ failure (SOFA) scores of 7.0 for control (placebo) patients, 7.7 for EPA treated, 6.7 for HMB and 8.1 for patients treated with both EPA and HMB. SOFA scores were not significantly different across the four groups. Patient groups also had significant chronic medical comorbidities, with mean Charlson Comorbidity Scores (CCS) of 2.9–4.0 for the four experimental groups. CCS were not different across the four groups. Patients had mean durations of previous mechanical ventilation of 7.3–9.2 days prior to study entry, and all had failed at least one attempt to wean from mechanical ventilation.

Several other patient characteristics are presented in Table 1. There were no significant differences in age, vital signs, mechanical ventilator settings, or lung mechanics (i.e. compliance and resistance) across the four experimental groups. The mean number of treatment doses that patients in the four groups received ranged from 17 to 19, indicating that there was good compliance with administration of medications to all groups of experimental subjects.

More detailed information, on a subject-by-subject basis, is provided on-line in the Additional file 2 (Detailed Subject Information.docx). This table provides diagnoses, activity levels prior to admission, and reason for admission for each participant in this study. Almost all patients were admitted for medical diagnoses, with a high percentage in all four groups having diagnoses of infections. The percentage of patients transferred to the MICU from other services was similar for the four groups. In addition, length of stay prior to ICU transfer was similar for the four groups, averaging means of 2.5 days (95% CI 0.42–4.48), 2.8 days (95% CI − 0.97 to 6.53), 3.5 days (95% CI 0.76–6.24), and 1.1 days (95% CI − 0.34 to 2.56), respectively, for Control, EPA, HMB, and HMB + EPA groups (*p* = 0.595).

### Physical therapy and caloric intake

**Fig. 2:**
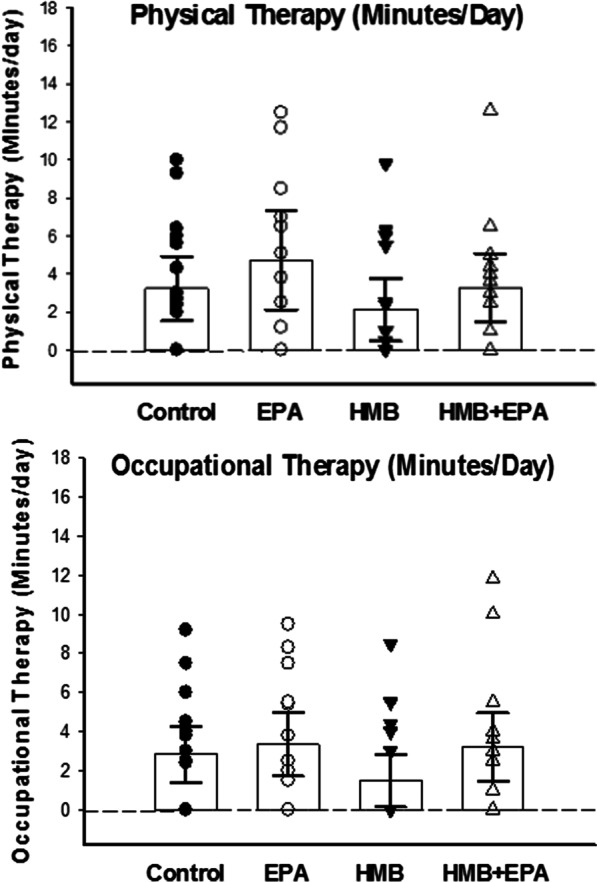
The average duration of physical therapy per day (top panel) and the average duration of occupational therapy per day (bottom panel) is presented for the four groups of patients included in the current study, i.e. placebo treated control patients, patients treated with EPA, patients treated with HMB, and patients treated with the combination of HMB and EPA. Both figures present data for individual subjects included in the four experimental groups. Group means for the experimental groups are presented as vertical bars (top of bar) and error bars indicate the 95% CI of the mean for the experimental groups. The mean duration of physical therapy per day was 3.23 min (95% CI 1.43–5.04), 4.72 min (95% CI 2.12–7.31), 2.12 min (95% CI 0.48–3.77), and 3.27 min (95% CI 1.48–5.06), respectively, for Control, EPA, HMB, and HMB + EPA groups (*p* = 0.278). The mean duration of occupational therapy per day was 2.8 min (95% CI 1.4–4.2), 3.1 min (95% CI 1.5–4.7), 1.5 min (95% CI 0.2–2.5), and 3.2 min (95% CI 1.5–4.9), respectively, for Control, EPA, HMB, and HMB + EPA groups (*p* = 0.267)

Average durations of physical and occupational therapy per day are provided in Fig. 2 for patients randomized to the four groups of treatments. Unfortunately, the ability of physical and occupational therapists to exercise patients in this study was limited by several factors, including medical instability, the requirement for off-unit diagnostic testing, and the need for time consuming ICU procedures. These events resulted in cancellation of planned therapy sessions, and as a result, the average combined durations of physical and occupational therapy achieved in the four groups (Fig. 2) were shorter than the goal of 10 min per day.

**Fig. 3:**
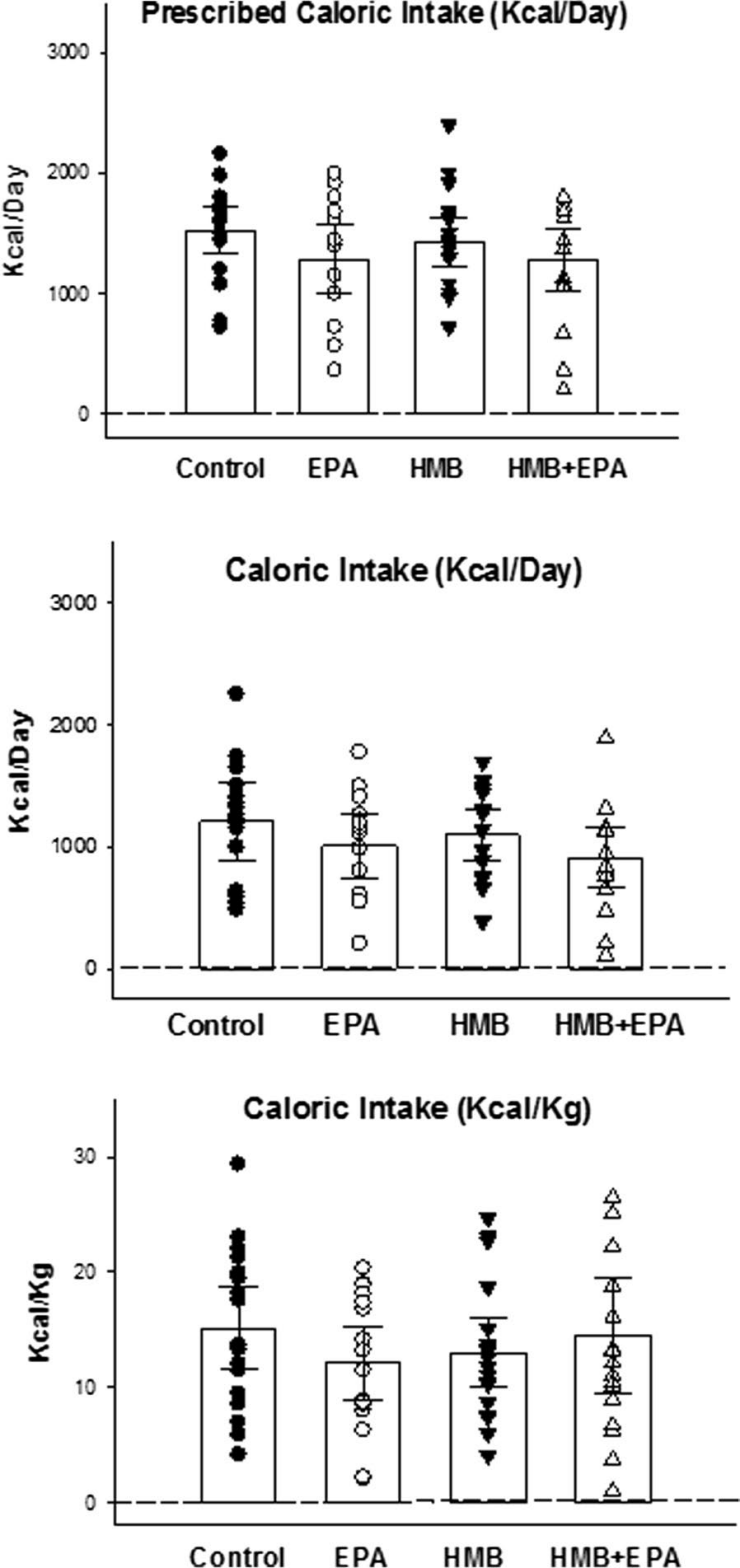
Prescribed caloric intakes, actual delivered caloric intakes, and calories/kg/day are presented for the four experimental groups. The prescribed caloric intake per day was 1526 kcal (95% CI 1325–1727), 1284 kcal (95% CI 994–1574), 1426 kcal (95% CI 1223–1629), and 1276 kcal (95% CI 1022–1530), respectively for Control, EPA, HMB, and HMB + EPA groups (*p* = 0.337). The mean caloric intake per day was 1207 kcal (95% CI 952–1462), 1010 kcal (95% CI 742–1279), 1096 kcal (95% CI 894–1298), and 912 kcal (95% CI 663–1160), respectively for Control, EPA, HMB, and HMB + EPA groups (*p* = 0.296). The mean caloric intake/Kg per day was 15.1 (95% CI 11.5–18.7), 12.1 (95% CI 8.9–15.3), 13.0 kcal (95% CI 10.0–16.0), and 14.5 kcal (95% CI 9.3–19.7), respectively for Control, EPA, HMB, and HMB + EPA groups (*p* = 0.641). As a result, there were no significant differences in caloric intake (Kcal/Day) or caloric intake normalized to body weight (Kcal/Kg) between the four groups

**Fig. 4:**
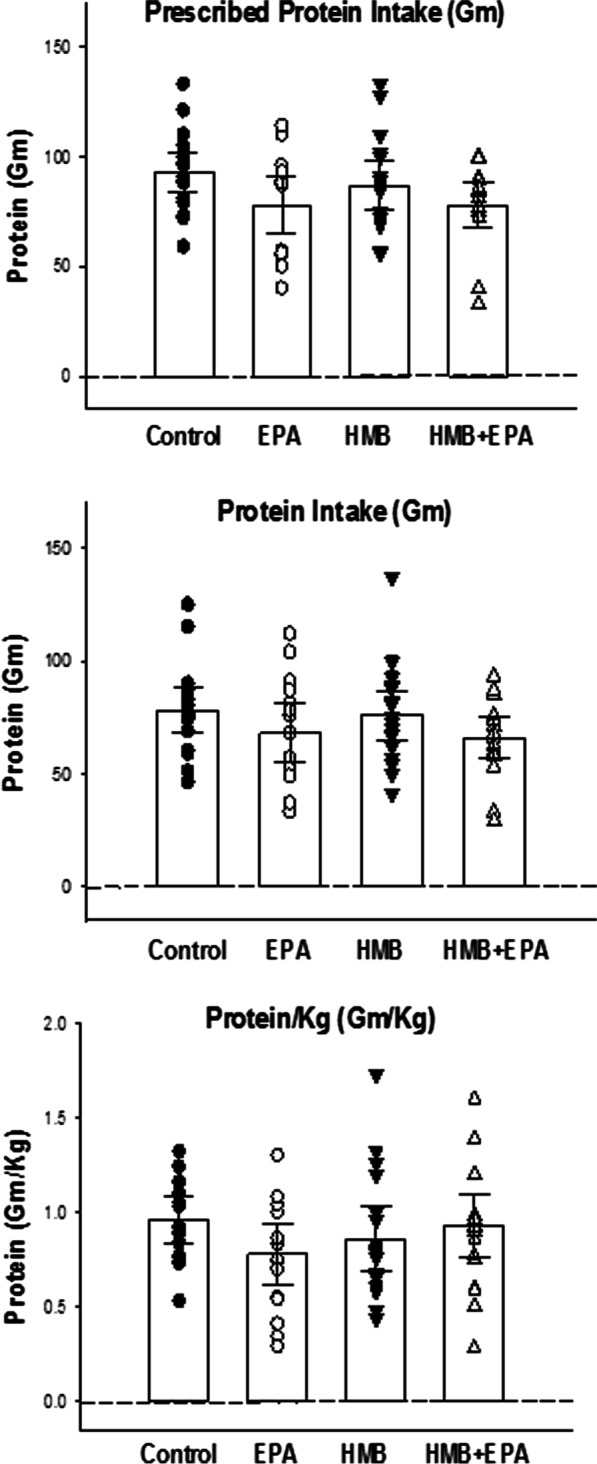
Prescribed protein intakes, actual delivered protein intakes, and protein/kg/day are presented for the four experimental groups. The prescribed protein intake in grams was 94 (95% CI 85–103), 78 (95% CI 65–91), 87 (95% CI 76–98), and 78 (95% CI 69–87), respectively for Control, EPA, HMB, and HMB + EPA groups (*p* = 0.086). The mean protein intake in grams was 78 (95% CI 68–88), 68 (95% CI 55–81), 76 (95% CI 65–87), and 66 (95% CI 57–75), respectively for Control, EPA, HMB, and HMB + EPA groups (*p* = 0.276). The mean protein intake in Gm/Kg was 0.96 (95% CI 0.84–1.08), 0.78 (95% CI 0.62–0.94), 0.86 (95% CI 0.69–1.03), and 0.92 (95% CI 0.75–1.09), respectively for Control, EPA, HMB, and HMB + EPA groups (*p* = 0.373). As a result, there were no significant differences in protein intake (Kcal/Day) or protein intake normalized to body weight (Kcal/Kg) between the four groups

All patients received enteral tube feedings and all patients also received protein supplements. The level of caloric and protein intakes were similar in all four treatment groups (Figs. 3 and 4) and were within the range found to produce acceptable outcomes in ICU patients [24].

### Diaphragm strength and thickness

**Fig. 5:**
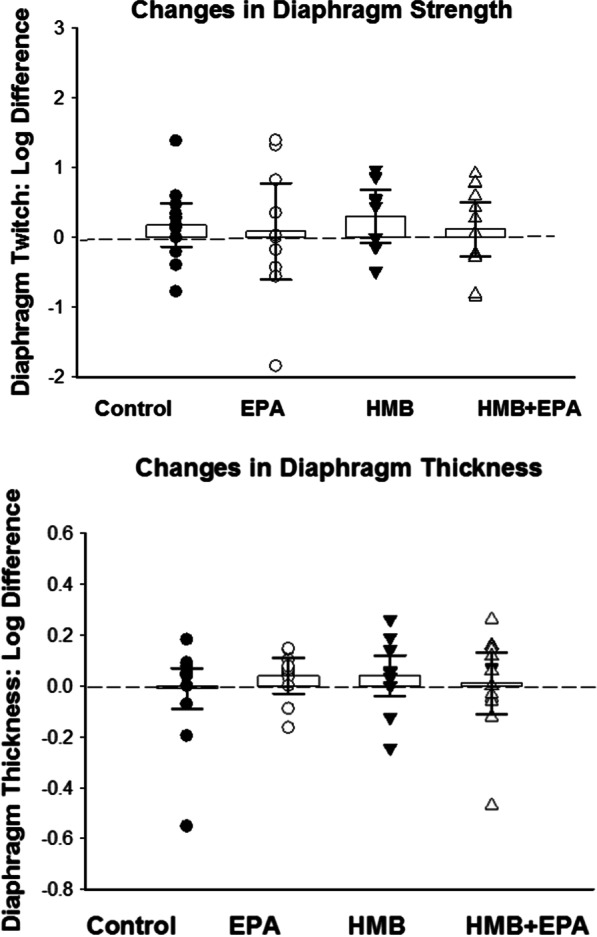
The change in diaphragm strength (PdiTw) between initial measurements and measurements made after drug treatment are presented for the four experimental groups in the top panel. Data is displayed as the log difference (*y*-axis) of pre- and post-treatment diaphragm twitch measurements for individual subjects included in the four experimental groups. The mean log difference for diaphragm twitch pressure (PdiTw) pre- and post-treatment was 0.18 (95% CI − 0.14 to 0.49), 0.09 (95% CI − 0.60 to 0.78), 0.31 (95% CI − 0.07 to 0.68), and 0.12 (95% CI − 0.27 to 0.50), respectively, for Control, EPA, HMB, and HMB + EPA groups (*p* = 0.894). As a result, there was no significant difference in change of PdiTw over time between the four experimental groups. Change in diaphragm thickness between initial and post-treatment measurements is presented for the four experimental groups in the bottom panel. The mean log difference for diaphragm thickness pre- and post-treatment was − 0.01 (95% CI − 0.10 to 0.09), 0.04 (95% CI − 0.03 to 0.10), 0.04 (95% CI − 0.04 to 0.13), and 0.01 (95% CI − 0.11 to 0.13), respectively, for Control, EPA, HMB, and HMB + EPA groups (*p* = 0.839). As a result, there was no significant difference in changes in diaphragm thickness over time between the four experimental groups

Patients had profoundly weak diaphragms at study entry, as shown in Table 2. Diaphragm strength, as assessed by measurement of diaphragm twitch generation (PdiTw) in response to bilateral supramaximal magnetic stimulation of the phrenic nerves (i.e. the BAMPS technique) was extremely low in the four experimental groups, with average levels less than 7 cm H_2_O in all four groups. This value is similar to that previously reported for MICU patients [1–4] but much lower than values observed in normal healthy subjects (which average 29 cm H_2_O, with a lower limit of normal of 15 cm H_2_O) [1, 21, 25]. ANCOVA analyses found no significant differences in adjusted mean changes between the four arms for either PdiTw (*p* = 0.89) or thickness (*p* = 0.50). Importantly, we found that PdiTw did not improve significantly in response to any of the therapeutic regimens (i.e. EPA, HMB, or HMB + EPA) employed in this study (Fig. 5). We also found that diaphragm thickness was similar in the four experimental groups at study initiation (Table 2). Moreover, diaphragm thickness did not increase in response to EPA, HMB or the combination of HMB + EPA (Fig. 5).

### Quadriceps strength and thickness

**Fig. 6:**
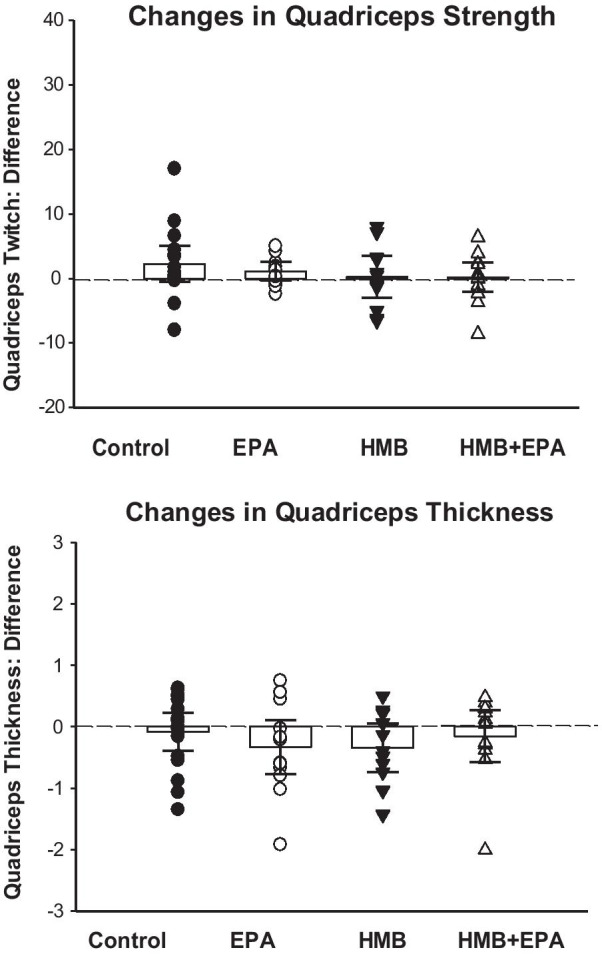
Change in quadriceps strength (QuadTw) between initial and post-treatment measurements is presented for the four experimental groups in the top panel (same format as in Fig. 5). The mean difference for quadriceps twitch force pre- and post-treatment was 2.26 (95% CI − 0.51 to 5.0), 1.12 (95% CI − 0.34 to 2.59), 0.26 (95% CI − 3.01 to 3.57), and 0.18 (95% CI − 2.07 to 2.43), respectively, for Control, EPA, HMB, and HMB + EPA groups (*p* = 0.535). As a result, there was no significant difference in changes in quadriceps strength over time between the four experimental groups. Change in quadriceps thickness between initial and post-treatment measurements is presented for the four experimental groups in the bottom panel. The mean difference for quadriceps twitch thickness pre- and post-treatment was − 0.08 (95% CI − 0.39 to 0.23), − 0.33 (95% CI − 0.77 to 0.11), − 0.34 (95% CI − 0.74 to 0.06), and − 0.15 (95% CI − 0.58 to 0.27), respectively, for Control, EPA, HMB, and HMB + EPA groups (*p* = 0.647). As a result, there was no significant difference in changes in quadriceps thickness over time between the four experimental groups

Patients also manifested severe quadriceps weakness at the time of recruitment into the current study. As shown in Table 2, the median quadriceps twitch force was less than 5 Newtons in all four treatment arms of the current study. In contrast, the quadriceps twitch force reported for healthy subjects in a recent study is several fold higher [22]. The initial quadriceps twitch force was similar, however, for patients in the four treatment groups of the present report. ANCOVA analyses found no significant differences in adjusted mean changes between the four arms for either quadriceps twitch force (*p* = 0.46) or thickness (*p* = 0.43). As a result, no treatments improved quadriceps twitch force over time or increased quadriceps thickness (Fig. 6).

### Duration of mechanical ventilation

**Fig. 7:**
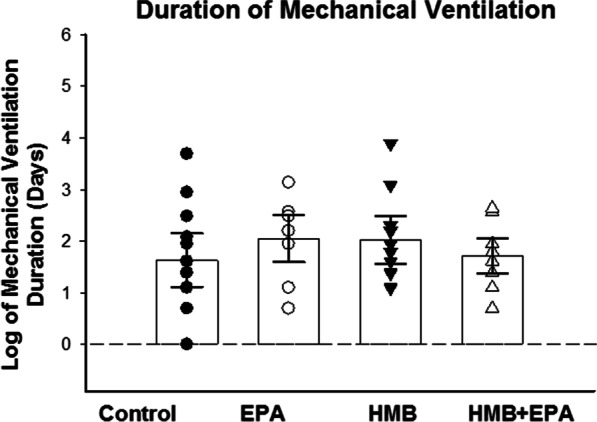
The duration of mechanical ventilation after randomization to treatment is presented for the four experimental groups using the format in Fig. 2. The mean log of mechanical ventilation duration was 1.63 (95% CI 1.10–2.15), 2.05 (95% CI 1.59–2.51), 2.03 (95% CI 1.57–2.49), and 1.71 (95% CI 1.38–2.05), respectively, for Control, EPA, HMB, and HMB + EPA groups (*p* = 0.372). There was no significant difference in the time required to wean patients from mechanical ventilation between the four experimental groups

As shown in Fig. 7, there was no significant difference in the time required to wean patients from mechanical ventilation across the experimental groups in this study. Specifically, neither EPA, HMB, or the combination of EPA + HMB reduced the duration of mechanical ventilation compared to placebo.

## Discussion

### Summary of major findings

The current study found evidence of profound weakness of both diaphragm and leg muscles at study entry, with both findings consistent with previous studies of muscle weakness in ICU patients [1–4, 7, 8]. The main finding of the present study, however, is that neither administration of beta-hydroxy-beta-methylbutyrate (HMB) nor eicosapentaenoic acid (EPA) or the combination of both agents increased either diaphragm or quadriceps muscle strength or muscle thickness in mechanically ventilated medical ICU patients. Specifically, we found that administration of 10 days of these anabolic agents did not increase either diaphragm PdiTw or quadriceps muscle QuadTw levels in this patient cohort. In addition, the duration of mechanical ventilation was not altered by administration of HMB, EPA or HMB + EPA, with all experimental groups requiring the same time to be successfully weaned from mechanical ventilator support.

### Previous studies using HMB and EPA

The findings of the present study are surprising, since several previous manuscripts have reported improvements in muscle strength in response to HMB and EPA in both cancer patients and in older adults [13–16]. In addition, HMB has been reported to appreciably reduce mortality when administered after discharge to patients with chronic lung and cardiac diseases [26]. One study also found that EPA administration to critically ill patients reduced the time required to wean patients from mechanical ventilation by 2 days [27]. In addition to these clinical reports, several animal studies have previously shown that HMB and EPA improve muscle function in animal models of illness [28, 29].

On the other hand, a recent study by Nakamura et al. [20] in which HMB was given to critical ill patients reported findings that are consistent with the findings in the present report. Specifically, Nakamura et al. randomized a group of critically ill patients to treatment with either routine nutrition or a nutritional regimen including 3 g/day of HMB, 14 g of arginine, and 14 g of glutamine daily for 10 days. These authors then examined the effects of these two nutritional regimens on femoral muscle volume assessed with computerized tomography. This previous report found that HMB/arginine/glutamine complex supplementation did not inhibit muscle volume loss in critically ill patients. The current report extends these findings demonstrating that 10 days of treatment with HMB also does not improve quadriceps strength and, in addition, does not improve diaphragm strength or thickness, in critically ill MICU patients.

### Potential role of physical therapy in modulating responses to HMB and EPA

There were, however, several limitations to the present study that may have compromised the ability of HMB and EPA to have salutary effects. All the patients in this study were to receive the standard paradigm of physical therapy applied to patients in the University of Kentucky intensive care units. The hospital protocol calls for patients to be evaluated within 48 h of ICU admission and to then receive physical therapy 3 days per week for the duration of their ICU stays. In addition, patients also received occupational therapy, which included exercises for muscles in the upper body. Physical and occupational therapy sessions were cancelled, however, when patients were deemed too unstable to tolerate treatments or if ongoing nursing, diagnostic or therapeutic efforts prevented patient exercise.

Because of these issues, only a small proportion of patients received anticipated daily durations of physical therapy (e.g. over 10 min of high quality physical and occupational therapy per day). In contrast, previous studies examining the effects of HMB and EPA in older adults and cancer patients included more intense exercise training as a component of the therapeutic regimen [13–16]. In addition, previous animal studies using these agents did not limit physical activity [28, 29]. It therefore seems possible that HMB and EPA require some level of concomitant leg exercise to exert beneficial effects on leg muscle function and the low levels of physical therapy our patients received limited the effectiveness of these drugs to improve quadriceps strength or thickness.

On the other hand, there is no evidence that limb muscle exercise has any effect on diaphragm strength or thickness in MICU patients. In addition, no previous study has examined the effects of either HMB or EPA on diaphragm strength or thickness in any patient population. As a result, there is also no reason to believe that reductions in the provision of physical therapy in the present study limited the effectiveness in HMB and/or EPA to improve diaphragm parameters.

### Potential role of timing and dosage of treatment with HMB and EPA

Another factor that may have compromised the ability of HMB and EPA to impact muscle function in the current study is the timing of drug administration. In previous studies employing animal models of disease (e.g. sepsis), HMB and EPA were administered soon after the induction of sepsis [28, 29]. These reports found that these two agents were capable of preventing the development of sepsis induced muscle dysfunction but did not determine if administration of these agents at late time points after the induction of weakness resulted in reversal of muscle dysfunction. There are reasons to believe, moreover, that the processes responsible for the induction of critical care related muscle dysfunction may be quantitatively and qualitatively different from the factors that result in a failure of muscles to recover [30]. For example, muscle protein synthesis declines early after the induction of sepsis but later increases to supra-normal levels [36]. HMB is known to prevent phosphorylation of eiF2α [31] and may thereby block the early sepsis induced reductions in protein synthesis, but, theoretically, this action should not be effective in improving muscle function if HMB is given well after muscle weakness has developed. In the present work, patients were often recruited into the study days after they became sick, with patients receiving an average of 6 days of mechanical ventilation before study entry. Moreover, the vast majority of patients were profoundly weak at the time of the initial measurements, again arguing that a significant time had elapsed between the insult inducing muscle injury and the time that therapies were given. It is possible that this delay prevented these agents from having beneficial effects. On the other hand, it is also important to point out that all the arguments presented in the previous paragraph are based on extrapolations from studies done in non-ICU patients or animals. The ideal timing of administration of HMB, EPA, or other anabolic agents to MICU patients is completely unknown.

One might also ask if a higher dose of HMB or EPA or a different route of administration of these agents might have elicited a positive response. The HMB and EPA doses used in this study are standard doses used in many previous studies in which these agents were found to have a beneficial effect on muscle function. While a few studies have used higher doses of HMB than 3 g/day, there is no convincing evidence that higher doses are more effective in improving muscle function. The enteral route of administration of these agents has also been used in the vast majority of human studies examining the effects of these agents on muscle [20]. In fact, a large percentage of enteral formulations used to feed ICU patients contain HMB, EPA, or both, and it has been assumed by major manufacturing companies that this route of administration is adequate to deliver these agents to achieve therapeutic effects. The current findings raise the possibility that these assumptions may be incorrect. It is possible that absorption of these agents may have been impaired in our extremely ill patient population, limiting the effectiveness of these agents to improve muscle function.

### Number of patients studied

The number of patients recruited into this study was based on a prior sample size calculation that estimated 18 patients per group, for a total of 72 patients enrolled, would be required to detect an increase in muscle strength in response to one of the treatments being studied (HMB or EPA). One might ask if a statistically significant response would have been detected had we studied more patients. Increasing the number of patients studied, however, would not be expected to change the magnitude of the increases observed over time in the various experimental groups or the variance of the strength measurements. Inspection of our data reveals that, at best, diaphragm twitch pressure increased by only 2 cm H20 in any of the four experimental groups and quadriceps twitch force increased only 2 Newtons in the four experimental groups after 10 days of therapy. We believe increments of this magnitude are insufficient to alter patient outcomes. One might argue that had we studied many more patients, we might have proven that these increments may be statistically significant, but they would remain clinically irrelevant.

### Persistence of quadriceps and diaphragm weakness

The failure of the therapies in the current study while disappointing, does not, however, diminish the importance of skeletal muscle weakness as a risk factor for poor outcomes in critically ill, mechanically ventilated patients. Previous work indicates that diaphragm twitch pressures less than 8 cm H_2_O are associated with an extremely high mortality and a protracted requirement for mechanical ventilation [1–4]. The vast majority of patients in the current study, i.e. 86%, had PdiTw levels less than 8 cm H_2_O at the time of study inclusion and therefore fell into this high-risk category. In addition, PdiTw levels did not increase significantly in any treatment group and at the end of study treatment administration (i.e. 10 days); 85% of all patients still manifested PdiTw levels less than 8 cm H_2_O. This finding indicates that diaphragm function did not substantially improve over time in any of the patients in the study and remained dangerously low. Similarly, the majority of patients had extremely low quadriceps twitch pressures, with 85% having a QuadTw value less than 6 Newtons at study inclusion. Like diaphragm function, quadriceps strength did not improve over time in any group and the majority of patients (85%) had QuadTw levels less than 6 Newtons after the period of study drug administration, i.e. 10 days.

### Implications for future clinical trials with anabolic agents

Physical therapy remains the one established approach to try to improve muscle function and patient outcomes in ICU patients. There are even limitations to this approach, and early intensive physical therapy, termed early mobilization, does not appear to benefit patients more than standard physical therapy [32].

Unfortunately, there are also no established adjunctive forms of therapy that are clearly capable of eliciting major improvements in the strength of either the quadriceps or diaphragm in critically ill patients. While some work suggests that use of NMES (neuromuscular electrical stimulation) may improve quadriceps strength, other studies indicate these effects are small [33–35]. In addition, while inspiratory muscle training has been used to improve diaphragm function in some studies of mechanically ventilated patients, other studies found this treatment did not improve outcomes [36, 37]. Diaphragm pacing is another form of treatment that has been postulated to increase diaphragm strength and improve outcomes, but this therapy will require substantial clinical trial testing before it can be assumed that this is an effective treatment [38, 39]. In addition, to our knowledge, there are no studies that have established the utility of administration of any pharmacological therapies to improve muscle strength in ICU patients.

The current findings, therefore, reinforce the need for additional therapeutic trials to prevent and reverse skeletal muscle dysfunction in critically ill patients. Importantly, the failure of the current study to identify a successful treatment provides some guidance for the design of future trials in order to avoid the pitfalls observed in the present report.

First, we believe additional basic science research is needed to more fully determine the cellular mechanisms by which disease processes reduce muscle function in the critically ill. Multiple processes interact to reduce muscle function in this population including systemic inflammation, inactivity, alterations secondary to nutritional deficiencies, and muscle damage due to aberrant contraction patterns. Therapies which may be effective in treating one of these processes (increased muscle contraction to counteract inactivity) may be ineffective in treating other processes (e.g. inflammation) or may worsen muscle function for other processes (e.g. aberrant muscle contraction). Second, based on current understanding, future clinical studies should mandate structured respiratory and limb muscle physical training regimens of at least moderate intensity as a component of treatment protocols. Third, it is reasonable to believe most anabolic agents should be administered at an early time point (e.g. within 48 h of the onset of critical illness) to prevent the potential irreversible loss of muscle reparative elements. Fourth, anabolic agents need to be developed that facilitate muscle recovery/repair for use in clinical trials studying patient populations with pre-existing severe muscle weakness. Identification of such agents and conduct of such future trials is urgently needed to develop an effective treatment to prevent the devastating acute and long-term consequences of critical care induced diaphragm and limb muscle dysfunction.

## Conclusions

These results indicate that HMB and EPA did not improve skeletal muscle strength in medical ICU patients when given as a 10 day course. This finding is mitigated, however, by the fact that many of the ICU patients in this study were too unstable to receive planned durations of daily physical therapy and it is possible that responses to medications may have been better if more physical therapy could have been provided. No one knows, however, exactly how much physical therapy is needed to improve the response of ICU patients to nutritional supplements. This important issue needs to be studied in detail in future clinical trials.

The findings of this study also confirm previous reports that diaphragm and leg strength of medical ICU patients are profoundly low, emphasizing the need for additional therapeutic trials to prevent and reverse skeletal muscle dysfunction in critically ill patients. It is hoped that the lessons learned from the current study will help investigators in the design of future clinical trials to examine the effects of other anabolic agents and innovative forms of physical therapy on skeletal muscle function in critically ill patients.

## Supplementary Information

**Additional file 1**. Detailed methods.

**Additional file 2**. Detailed subject information.

## Data Availability

Summaries of the datasets generated and analyzed during the current study are available for review at ClinicalTrials.gov and can be accessed at the following address: https://clinicaltrials.gov/ct2/show/NCT01270516?term=Supinski&draw=2&rank=4. The raw datasets used and/or analyzed during the current study are available from the corresponding author on reasonable request.

## Abbreviations

ICU: Intensive care unit
HMB: Hydroxymethylbutyrate
EPA: Eicosapentaenoic acid
PdiTw: Transdiaphragmatic twitch pressure
QuadTw: Quadriceps twitch force
ANOVA: Analysis of variance
IRB: Institutional review board
FiO_2_: Fraction of inspired oxygen
H_2_O: Water
PEEP: Positive end expiratory pressure
BAMPS: Bilateral anterior magnetic phrenic stimulation
MHz: Megahertz
VIM: Vastus intermedius
RF: Rectus femoris
SOFA: Sequential organ failure score
CCS: Charlson comorbidity index score
NMES: Neuromuscular electrical stimulation
CI: Confidence interval
PT: Physical therapy
OT: Occupational therapy
