# Supplementary material for: A randomized controlled trial to determine whether beta-hydroxy-beta-methylbutyrate and/or eicosapentaenoic acid improves diaphragm and quadriceps strength in critically Ill mechanically ventilated patients
Source: Crit Care. 2021 Aug 26;25:308. doi: 10.1186/s13054-021-03737-9 (PMC8390080; doi:10.1186/s13054-021-03737-9)
Supplement: Supplementary file 1 — Additional file 1. Detailed methods. [file 13054_2021_3737_MOESM1_ESM.docx]

**Additional Methods**

**Nutrition Protocol**

The following protocol was used to provide tube feeds to study patients (protocol developed in concert with Barbara Magnuson, head of the MICU nutritional team at the University of Kentucky):

(1) First, basal energy expenditure (i.e. caloric requirements) was estimated based on the Harris- Benedict equations:

BEE Men = 66 + (13.7 x Wt kg) + (5.0 x Ht cm) – (6.8 x Age yrs)

BEE Women = 665 + (9.6 x Wt kg) + (1.7 x Ht cm) – (4.7 x Age yrs)

(2) Second, adjustment was made for obesity if >125% ideal body weight by adding 25% of the difference between BEE for actual and ideal body weight to the BEE for ideal body weight.

(3) Third, a multiplier was used to adjust caloric requirements to adjust for severity of illness; 1.2 for stroke, 1.3 for pneumonia or ARDS

(4) Fourth, protein needs for unstressed patients were estimated as 0.8-1.0 gm/kg, for mild stress as 1.0-1.2 gm/kg, for moderate stress as 1.2-1.5 gm/kg, and for infection or severe stress as 1.3-1.6 gm/kg. Actual body weight was used unless >125% of ideal body weight in which case adjusted body weight will be used, as calculated in (2)

(5) Fifth, additional adjustments in nutrition were made for patients with renal or liver failure based on the University of Kentucky Adult Nutrition Support Handbook

(6) Sixth, an enteral feeding product was chosen. For the present study, we often used Jevity 1.2 which is composed of 18.5% protein calories, 29% fat calories, and 52.5% carbohydrate calories. For Jevity 1.2, the total calories required per day was calculated in (3) and was divided by 1.2 cal/ml to derive the volume of Jevity 1.2 to be provided over 24 hours. Of note, patients receiving Juven were not enrolled in the study as this product contains HMB (it was not the usual practice of MICU physicians at UK to use this formula). We also did not study any patients fed any other tube feed formulations containing either EPA or HMB.

(7) Once the Jevity 1.2 volume was determined, the protein provided by this delivery was calculated; additional protein to reach the daily protein needs of the patient was provided by adding Beneprotein supplement to bring the total protein delivery to that calculated in (4).

(8) Enteral access was established, if not already present, as per UK Enteral Access Guidelines

(9) The volume of enteral feeding was administered as a continuous feed delivered by a pump over 24 hours.

**Physical Therapy Protocol**

Subjects received the standard, conventional forms of physical therapy (PT) employed at the University of Kentucky for MICU patients, with the specific exercise paradigm used for each individual patient chosen by the facility staff. The standard PT protocol included: (a) an initial assessment session evaluating the patient’s medical condition including diagnoses and level of consciousness, previous level of functioning, ability to perform standard PT maneuvers, ability to perform activities of daily living, transfer and movement, an assessment of the strength of individual muscle groups, and development of an exercise prescription that included the types of exercises to be performed during sessions, and (b) subsequent exercise sessions were then conducted with a goal of three PT sessions per week with each session lasting 20-40 minutes. Typical components of therapy sessions included balance training, bed mobility training, gait training, lumbar stabilization, manual therapy techniques, motor coordination training, neuromuscular re-education, postural re-education, range of motion, stretching, transfer training, and strengthening exercises. Therapists recorded the modes of exercise delivered for PT sessions as well as the session duration. Sessions were cancelled if patients were deemed too unstable for PT (e.g. requiring 100% oxygen), had left the MICU to get diagnostic testing when the therapists arrived, or were undergoing medical procedures (e.g. central line placement).

**Patient Sedation**

All patients included in this study were receiving mechanical ventilation when first enrolled in the project. These patients were assessed for level of sedation/agitation, pain, and delirium daily and objective scores (including the Richmond Agitation Sedation Scale, RASS) for these parameters were reviewed daily during multidisciplinary rounds. The MICU practice team then made decisions about choices and levels of medications for sedation, pain, and delirium based on the patient course and condition. The most commonly used medication for sedation during the implementation of this project was propofol, typically administered as a continuous infusion and titrated to attain a targeted RASS level. Dexmedetomidine hydrochloride was also used for sedation, almost always for patients requiring less than 72 hours of treatment. The most common drug used to treat pain was fentanyl, typically administered as an infusion with a range of 25 – 50 mcg/hr. Patients rarely received benzodiazepines as a treatment, with the exception of patients with delirium tremens, for whom this class of drugs was uniformly administered.

**Ventilator Separation Protocol**

The University of Kentucky Ventilator Separation Protocol was employed in the University of Kentucky adult intensive care units at the time of this study and provided a defined approach to weaning patients from mechanical ventilation. Every morning mechanically ventilated patients were evaluated for potential weaning trial employment by respiratory therapy. Inclusion criteria for a spontaneous breathing trial (SBT) included evidence that the patient was: (a) awake, (b) had a gag reflex, (c) had a cough reflex, (d) had a RASS scale of -1 to 0, (e) had a FiO2 ≤50% and PEEP applied ≤ 6 H2O, (f) had a pH ≥7.35, (g) had a MAP of ≥ 65 mm Hg, (h) was off pressor drips (e.g. epinephrine) save for doses of dopamine ≤ 5 mcg/kg/min or dobutamine ≤ 5 mcg/kg/min, and (i) was not on an intra-aortic balloon pump. Exclusion criteria include increased intracranial pressure, use of paralytics, active seizures, excessive agitation and active myocardial ischemia. If the patient met all inclusion criteria and had no exclusion criteria, sedation was reduced and the patient received a spontaneous breathing trial (SBT) for 1 hour, with an arterial blood gas drawn at the end of the trial. Trials were stopped if any of the following events occurred: (a) oxygen saturation fell below 90% for more than three minutes, (b) the heart rate increased above 130 or fell below 60, (c) systolic blood pressure fell below 90 or increased above 180, (d) the mixed venous oxygen saturation fell below 60 mm Hg (if monitored), (e) the respiratory rate increased above 35 or the RSBI increased above 100, (f) the patient developed chest pain or a new dysrhythmia, (g) the patient’s mentation worsened, or (h) the patient developed diaphoresis, distress or excessive anxiety. Blood gases were examined at the end of trials and trials were judged a success if, in addition to passing the criteria in the preceding sentence, the final paO2 was greater than 60 mm Hg and there was no respiratory acidosis. If the patient achieved a successful SBT, the attending was then asked for an extubation order. If the trial failed, the patient was placed on their previous ventilator mode and settings. If trial failure was associated with evidence of excessive sedation, sedation levels were reduced in preparation for the next day trial.
