# Supplementary material for: A randomized controlled trial to determine whether beta-hydroxy-beta-methylbutyrate and/or eicosapentaenoic acid improves diaphragm and quadriceps strength in critically Ill mechanically ventilated patients
Source: Crit Care. 2021 Aug 26;25:308. doi: 10.1186/s13054-021-03737-9 (PMC8390080; doi:10.1186/s13054-021-03737-9)
Supplement: Supplementary file 2 — Additional file 2. Detailed subject information. [file 13054_2021_3737_MOESM2_ESM.docx]

**Table 1: Diagnoses, Prior Activity, Reason for Admission**

| Study Arm | Diagnoses | Activities  Before Admission | Admission Type | Reason for Admission | Hospital Days  Before ICU  Transfer |
| --- | --- | --- | --- | --- | --- |
| Control-1 | Sepsis,  Respiratory Failure | Independent ADL, transfer, ambulation | Medical | Respiratory failure | 0 Days |
| Control-2 | Sepsis,  Heart failure | Assistance, Wheelchair | Medical | Heart failure | 16 Days |
| Control-3 | Cystic fibrosis,  hemoptysis | Assistance, Walker | Medical | Respiratory failure | 0 Days |
| Control-4 | Seizure, Sepsis | Assistance, Device | Medical | Respiratory failure | 0 Days |
| Control-5 | Sepsis, Pneumonia | Independent ADL, transfer, ambulation | Medical | Respiratory failure | 0 Days |
| Control-6 | Sepsis, Cirrhosis | Assistance, Device | Medical | Respiratory failure | 0 Days |
| Control-7 | Pneumonia,  Asthma | Independent ADL, transfer, ambulation | Medical | Respiratory failure | 2 Days |
| Control-8 | Pneumonia  Heart failure | Independent ADL, transfer, ambulation | Medical | Heart failure | 9 Days |
| Control-9 | Pneumonia, Respiratory failure | Independent ADL, transfer, ambulation | Medical | Respiratory failure | 0 Days |
| Control-10 | Respiratory failure, Pneumonia | Independent ADL, transfer, ambulation | Medical | Pneumonia | 0 Days |
| Control-11 | Respiratory failure, Pneumonia | Assistance, Walker | Medical | Diabetes | 9 Days |
| Control-12 | Respiratory failure, Pneumonia | Assistance, Walker | Medical | Respiratory failure | 0 Days |
| Control-13 | Pneumonia, Respiratory failure | Independent ADL, transfer, ambulation | Medical | Pneumonia | 0 Days |
| Control-14 | COPD, Pneumonia | Independent ADL, transfer, ambulation | Medical | Respiratory failure | 0 Days |
| Control-15 | Shock,  Sepsis | Independent ADL, transfer, ambulation | Medical | Sepsis | 0 Days |
| Control-16 | Pneumonia, Sepsis | Assistance, Device | Medical | Respiratory failure | 0 Days |
| Control-17 | Pneumonia, Sepsis | Assistance,  Wheelchair | Medical | Graft rejection | 2 Days |
| Control-18 | Sepsis | Independent  ADL, transfer, ambulation | Surgery | Abscess | 1 Day |
| Control-19 | Pneumonia | Assistance, cane | Surgery | Clot removal | 6 Days |
| Control-20 | Respiratory failure | Independent | Surgery | Hysterectomy | 4 Days |

| Study Arm | Diagnoses | Activities  Before Admission | Admission Type | Reason for Admission | Hospital Days  Before ICU  Transfer |
| --- | --- | --- | --- | --- | --- |
| EPA-1 | COPD, Pneumonia | Independent ADL, transfer  Cane | Medical | COPD | 0 Days |
| EPA-2 | Sepsis,  Seizures | Assistance | Medical | Respiratory failure | 0 Days |
| EPA-3 | Sepsis, Pneumonia | Independent  ADL, transfer, ambulation | Medical | ARDS | 0 Days |
| EPA-4 | Heart failure, Respiratory failure | Assistance,  cane | Medical | Respiratory  failure | 1 Day |
| EPA-5 | Pneumonia,  Respiratory failure | Assistance,  Cane | Medical | Respiratory failure | 0 Days |
| EPA-6 | Sepsis, Respiratory failure | Independent  ADL, transfer, ambulation | Medical | Respiratory failure | 0 Days |
| EPA-7 | Sepsis  Respiratory failure | Independent  ADL, transfer, ambulation | Medical | Sepsis | 14 Days |
| EPA-8 | Cryptogenic organizing pneumonia | Independent  ADL, transfer, ambulation | Medical | Respiratory failure | 0 Days |
| EPA-9 | ARDS, Sepsis | Independent  ADL, transfer, ambulation | Medical | Respiratory failure | 0 Days |
| EPA-10 | Pneumonia,  Respiratory failure | Independent  ADL, transfer, ambulation | Medical | Respiratory failure | 0 Days |
| EPA-11 | Respiratory failure  Pneumonia | Independent  ADL, transfer, ambulation | Medical | Pneumonia | 0 Days |
| EPA-12 | Mycobacterium avian  intracellular | Assistance | Medical | Respiratory failure | 2 Days |
| EPA-13 | Respiratory failure, Pneumonia | Assistance, Walker | Medical | Cirrhosis | 1 Day |
| EPA-14 | Sepsis, Ventricular tachycardia | Assistance, Walker | Medical | Respiratory failure | 0 Days |
| EPA-15 | Pneumonia | Independent  ADL, transfer, ambulation | Medical | Pneumonia | 0 Days |
| EPA-16 | Pneumonia  Respiratory failure | Assistance | Medical | Fluid overload | 30 Days |
| EPA-17 | Sepsis | Independent  ADL, transfer, ambulation | Medical | Dyspnea | 1 Day |

| Study Arm | Diagnoses | Activities  Before Admission | Admission Type | Reason for Admission | Hospital Days  Before ICU  Transfer |
| --- | --- | --- | --- | --- | --- |
| HMB-1 | Pneumonia, Pulmonary edema | Assistance | Medical | Respiratory failure | 0 Days |
| HMB-2 | Congestive heart failure | Assistance, Walker | Medical | Respiratory failure | 0 Days |
| HMB-3 | Sepsis,  Empyema | Independent  ADL, transfer, ambulation | Medical | Respiratory failure | 0 Days |
| HMB-4 | COPD, Sepsis | Independent  ADL, transfer, ambulation | Medical | Respiratory failure | 1 Day |
| HMB-5 | Sepsis, Pneumonia | Independent  ADL, transfer, ambulation | Medical | Sepsis | 2 Days |
| HMB-6 | Sepsis, Respiratory failure | Assistance, Cane | Medical | Respiratory failure | 0 Days |
| HMB-7 | Sepsis, Respiratory failure | Assistance, Cane | Medical | Respiratory failure | 2 Days |
| HMB-8 | Sepsis, COPD | Independent  ADL, transfer, ambulation | Medical | Respiratory failure | 2 Days |
| HMB-9 | Respiratory failure, COPD | Assistance,  Cane | Medical | COPD | 1 Day |
| HMB-10 | Pneumonia, Respiratory failure | Assistance | Medical | Delirium tremens | 9 Days |
| HMB-11 | Pneumonia, Sepsis | Independent  ADL, transfer, ambulation | Medical | Hepatic encephalopathy | 9 Days |
| HMB-12 | COPD, Respiratory failure | Independent  ADL, transfer, ambulation | Medical | Respiratory failure | 0 Days |
| HMB-13 | Interstitial lung disease | Independent  ADL, transfer, ambulation | Surgical | Hysterectomy | 12 Days |
| HMB-14 | Microscopic polyangiitis | Independent  ADL, transfer, ambulation | Medical | Microscopic polyangiitis | 20 Days |
| HMB-15 | Pneumonia, Respiratory failure | Independent  ADL, transfer, ambulation | Medical | Respiratory failure | 0 Days |
| HMB-16 | Sepsis, Respiratory failure | Independent  ADL, transfer, ambulation | Medical | Airway obstruction | 1 Days |
| HMB-17 | Pneumonia, Respiratory failure | Ambulates with assistance | Medical | Respiratory failure | 0 Days |
| HMB-18 | Heart failure, Respiratory failure | Independent  ADL, transfer, ambulation | Medical | Hypoglycemia | 4 Days |

| Study Arm | Diagnoses | Activities  Before Admission | Admission Type | Reason for Admission | Hospital Days  Before ICU  Transfer |
| --- | --- | --- | --- | --- | --- |
| HMB-EPA-1 | GI bleeding, Pneumonia | Independent  ADL, transfer, ambulation | Medical | Pneumonia | 2 Days |
| HMB-EPA-2 | Sepsis, Pneumonia | Independent  ADL, transfer, ambulation | Medical | Respiratory failure | 1 Days |
| HMB-EPA-3 | ARDS, Sepsis | Independent  ADL, transfer, ambulation | Medical | Respiratory failure | 0 Day |
| HMB-EPA-4 | ARDS, Respiratory failure | Independent  ADL, transfer, ambulation | Medical | Respiratory failure | 0 Days |
| HMB-EPA-5 | Pneumonia, Sepsis | Assistance, Walker | Medical | Respiratory failure | 0 Days |
| HMB-EPA-6 | Sepsis, Respiratory failure | Independent  ADL, transfer, ambulation | Medical | Respiratory failure | 0 Days |
| HMB-EPA-7 | Sepsis, Respiratory failure | Assistance, Cane | Medical | Sepsis | 1 Day |
| HMB-EPA-8 | ARDS,  Heart failure | Independent  ADL, transfer, ambulation | Medical | Heart failure | 12 Days |
| HMB-EPA-9 | Pneumonia, Sepsis | Independent  ADL, transfer, ambulation | Medical | Respiratory failure | 0 Days |
| HMB-EPA-10 | Coal Workers Lung Disease, COPD | Independent  ADL, transfer, ambulation | Medical | Respiratory failure | 0 Days |
| HMB-EPA-11 | Sepsis, Pneumonia | Independent  ADL, transfer, ambulation | Medical | Respiratory failure | 0 Days |
| HMB-EPA-12 | Renal failure,  Respiratory failure | Independent  ADL, transfer, ambulation | Medical | Pancreatitis | 0 Days |
| HMB-EPA-13 | Sepsis, Respiratory failure | Assistance, Walker | Medical | Respiratory failure | 0 Days |
| HMB-EPA-14 | Heart failure, Respiratory  failure | Independent  ADL, transfer, ambulation | Medical | Seizures | 0 Days |
| HMB-EPA-15 | Pneumonia, Sepsis | Assistance | Medical | ARDS | 0 Days |
| HMB-EPA-16 | Sepsis, Respiratory failure | Independent  ADL, transfer, ambulation | Medical | Respiratory failure | 0 Days |
| HMB-EPA-17 | Respiratory failure | Assistance, Cane | Medical | Hemoptysis | 0 Days |
| HMB-EPA-18 | Respiratory  Failure, Pneumonia | Assistance, Walker | Medical | Fever | 4 Days |
